# Infantile status epilepticus disrupts myelin development

**DOI:** 10.1016/j.nbd.2021.105566

**Published:** 2021-11-24

**Authors:** Petra Bencurova, Hanne Laakso, Raimo A. Salo, Ekaterina Paasonen, Eppu Manninen, Jaakko Paasonen, Shalom Michaeli, Silvia Mangia, Martin Bares, Milan Brazdil, Hana Kubova, Olli Gröhn

**Affiliations:** a CEITEC - Central European Institute of Technology, Masaryk University, Kamenice 5, 625 00 Brno, Czech Republic; b Department of Neurology, St. Anne’s University Hospital and Medical Faculty of Masaryk University, Pekarska 53, 656 91 Brno, Czech Republic; c A.I. Virtanen Institute for Molecular Sciences, University of Eastern Finland, PO Box 1627, FI-70211 Kuopio, Finland; d Center for Magnetic Resonance Research, University of Minnesota, Minneapolis, MN, United States; e Department of Neurology, School of Medicine, University of Minnesota, Minneapolis, MN, United States; f Academy of Sciences Czech Republic, Institute of Physiology, Department of Developmental Epileptology, Videnska 1083, 14220 Prague, Czech Republic

**Keywords:** Animal model, Status Epilepticus, Temporal Lobe Epilepsy, Myelin development, White matter integrity, MRI, Histology, Thalamocortical connectivity

## Abstract

Temporal lobe epilepsy (TLE) is the most prevalent type of epilepsy in adults; it often starts in infancy or early childhood. Although TLE is primarily considered to be a grey matter pathology, a growing body of evidence links this disease with white matter abnormalities. In this study, we explore the impact of TLE onset and progression in the immature brain on white matter integrity and development utilising the rat model of Li-pilocarpine-induced TLE at the 12th postnatal day (P). Diffusion tensor imaging (DTI) and Black-Gold II histology uncovered disruptions in major white matter tracks (corpus callosum, internal and external capsules, and deep cerebral white matter) spreading through the whole brain at P28. These abnormalities were mostly not present any longer at three months after TLE induction, with only limited abnormalities detectable in the external capsule and deep cerebral white matter. Relaxation Along a Fictitious Field in the rotating frame of rank 4 indicated that white matter changes observed at both timepoints, P28 and P72, are consistent with decreased myelin content. The animals affected by TLE-induced white matter abnormalities exhibited increased functional connectivity between the thalamus and medial prefrontal and somatosensory cortex in adulthood. Furthermore, histological analyses of additional animal groups at P15 and P18 showed only mild changes in white matter integrity, suggesting a gradual age-dependent impact of TLE progression. Taken together, TLE progression in the immature brain distorts white matter development with a peak around postnatal day 28, followed by substantial recovery in adulthood. This developmental delay might give rise to cognitive and behavioural comorbidities typical for early-onset TLE.

## 1. Introduction

Epilepsy is a disease characterised by recurrent seizures that often affects all aspects of a patient’s life (Behr et al., 2016). Temporal lobe epilepsy (TLE), which is the most common type of epilepsy in adults, often starts in infancy or early childhood. The early onset of epilepsy, along with early life seizures, has adverse effects on a patient’s health beyond the manifestation of seizures: it can lead to cognitive impairment, epilepsy aggravation (seizure frequency, risk of mortality, etc.), morphological changes in the brain, and the development of specific comorbidities, including attention deficit hyperactivity disorder (ADHD), verbal memory and learning impairments, etc. (Hermann et al., 2002; Nickels et al., 2016; Sillanpää and Shinnar, 2010).

Despite epilepsy being predominantly considered a disease of grey matter, changes in white matter have been detected in both epileptic patients and animal models (Gross, 2011; Sierra et al., 2011; Urbach et al., 2017; Ye et al., 2013). Advanced magnetic resonance imaging (MRI) techniques allow for noninvasive high-resolution detection of white matter structures directly in the living brain. White matter alterations are most often identified by diffusion tensor imaging (DTI), which detects the magnitude and direction of restricted water diffusion in tissue, allowing MRI to be sensitised to microstructural changes in myelinated axons (Mori and Zhang, 2006). DTI has identified white matter disruption across the corpus callosum, cingulum, thalamic tracts, and temporal lobe, including hippocampal formation in TLE patients (Gross, 2011; Kimiwada et al., 2006; Liu et al., 2016; Urbach et al., 2017). Along with DTI, histological analyses have confirmed white matter disruption in the corpus callosum, fornix fimbriae, thalamus, and hippocampus in animal models of TLE (Salo et al., 2017; Sierra et al., 2011; van Eijsden et al., 2011; Ye et al., 2013).

While DTI is highly sensitive to microstructural changes and thus the most commonly used technique for white matter investigations, other MRI methods are sensitive to white matter changes, predominantly measuring interactions between water and myelin (Bock et al., 2009; Mackay et al., 1994). Among these, magnetisation transfer (MT) detects myelin loss in white matter fibres by detecting the transfer in magnetisation between macromolecules and water molecules following off-resonance saturation (Does et al., 1998; Mottershead et al., 2003). There is some ambiguity regarding the interpretation of MT data in the context of myelin loss (Mottershead et al., 2003; Newbould et al., 2014), which can be resolved by increasing MT specificity through the on-resonance inversion pulse before off-resonance irradiation (Mangia et al., 2011). The recently developed Relaxation Along a Fictitious Field in the rotating frame of rank n (RAFFn) method allows the detection of slow (up to millisecond time scale) motions by detecting relaxation time constants in the presence of fictitious fields generated by the modulation of radiofrequency pulses (Liimatainen et al., 2010). RAFF sensitivity includes the millisecond range of the correlation times, which likely corresponds to the conformational exchange of myelin-associated methylene groups (Hakkarainen et al., 2016; Satzer et al., 2015). Higher rank RAFFn techniques (RAFF4, RAFF5) provide higher tissue contrast between grey and white matter and, relative to other methods, increase sensitivity to demyelination and dysmyelination in tracts with heterogeneous fibre orientations or multidirectional white matter disruption (Hakkarainen et al., 2016; Holikova et al., 2021; Liimatainen et al., 2015; Satzer et al., 2015).

White matter integrity is crucial for proper signal transduction within neuronal networks, and its disruption leads to changes in neuronal wiring and excitability (Seidl, 2014). Brain areas involved in the same network show synchronous activity and metabolic needs that correlate with changes in the blood oxygenation level dependent (BOLD) signal in functional MRI (fMRI). Resting-state fMRI provides information about baseline functional neuronal connectivity with the potential to detect pathological abnormalities (Greicius, 2008). Changes in neuronal connectivity manifest themselves in large networks (e.g. default mode networks) in TLE, both involved and independent of connections associated with the epileptic onset zone (Centeno and Carmichael, 2014; Haneef et al., 2014). The thalamus is often connected to these networks as a principal relay site for the majority of projections in the brain (Cunningham et al., 2017; Doucet et al., 2013; Haneef et al., 2014; Holmes et al., 2013). The thalamus has been implicated in seizure propagation and the modulation of the motor component of seizures in animal models (Gale, 1992).

White matter integrity and brain connectivity are modulated by ongoing TLE. However, in the immature brain, the effect of TLE onset on white matter development remains unknown. As compared with late-onset cases, patients with childhood-onset TLE show a smaller white matter volume that correlates with deteriorated cognitive performance (Hermann et al., 2002). It is unknown whether these white matter alterations occur as a direct outcome of epileptogenesis onset in the immature brain or due to medical interventions during the critical period of white matter development.

In this study, we investigated white matter integrity in TLE with onset in infancy. We induced TLE-like syndrome by LiCl-pilocarpine status epilepticus (SE) in rat pups at postnatal day 12 (P12). To address the integrity of white matter during (P28) and after its development (3 months) within the same group of animals, we used MRI techniques with high sensitivity to alterations in myelin integrity and content, namely DTI, MT, and RAFF4 quantitative mapping. Following the three-month white matter imaging time-point, we assessed resting-state functional connectivity with a focus on thalamic connectivity.

## 2. Materials and methods

### 2.1. Animals

Male Wistar albino rats (*n* = 76) were used in this study (RccHan: WIST, Kuopio Laboratory Animal Centre, Kuopio, Finland & Institute of Physiology, Czech Academy of Sciences, Prague). The day of birth was defined as day P0, and the animals were weaned on P28. All animal procedures were approved by the Animal Health and Welfare committee of the Regional State Administrative Agency (Approval No. ESAVI/270/04.10.07/2017) or by the Ethical Committee of the Czech Academy of Sciences (Approval No. 128/2013) and conducted following the guidelines set by the European Commission Directive 2010/63/EU for animal experiments and ARRIVE guidelines (Kilkenny et al., 2010; Percie du Sert et al., 2020). The animals were housed in a controlled environment (22 ± 2 °C; 50–60% humidity) with a 12-h light/dark cycle. Food and water were provided ad libitum.

### 2.2. Induction of epileptic status

**Fig. 1.**
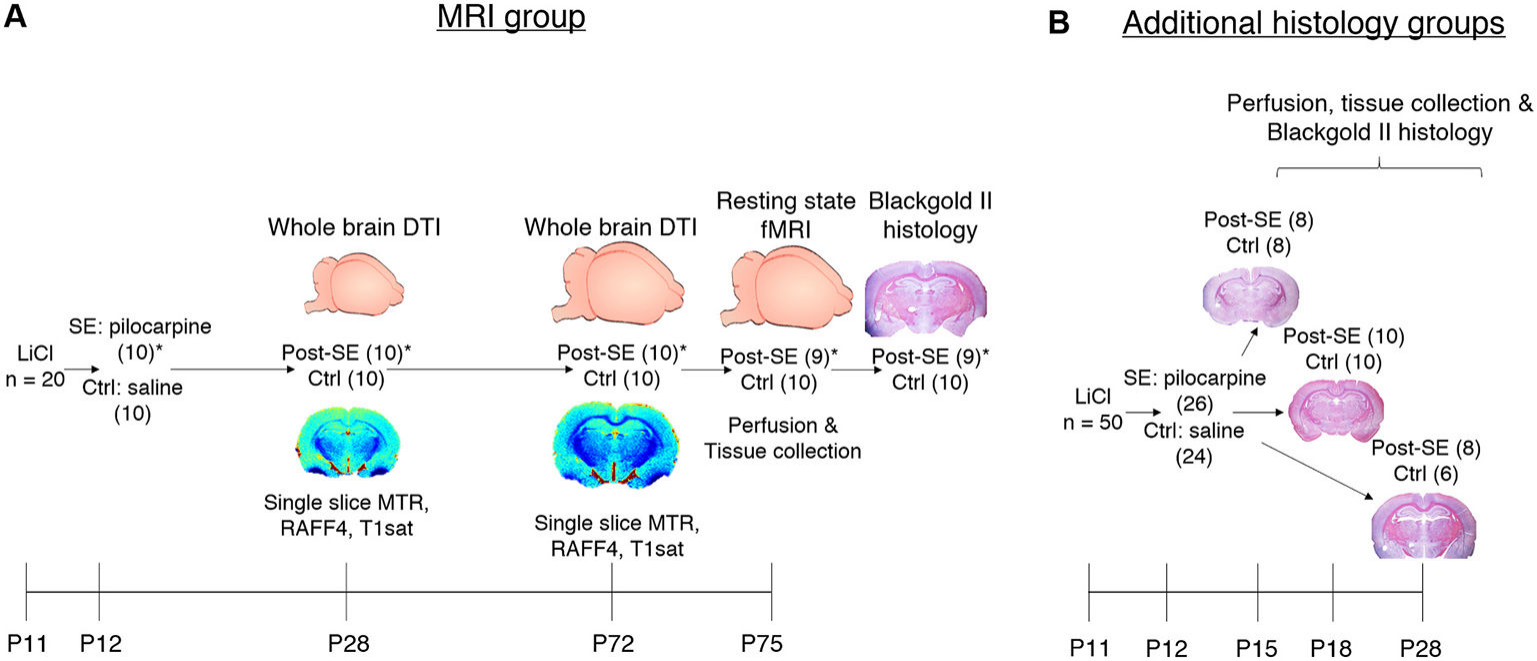
Study outline. The figure describes the experimental procedures performed in each animal group: MRI group (**A**) and Additional histology groups (**B**) at respective age represented by the number of postnatal days (P). White matter integrity was compared at given age between rats with status epilepticus (SE) induced at P12 and controls (Ctrl) across the whole brain combining diffusion tensor imaging (DTI) and histology. Regions of interest (ROIs) were analysed in a single slice at a position −3.2 mm from the bregma in the coronal plane by a battery of white matter sensitive MRI techniques: Magnetisation transfer ratio (MTR), Relaxation Along a Fictitious Field (RAFF4), and T1 relaxation time under magnetisation transfer irradiation (T_1sat_). The numbers of rats per group are displayed in parentheses. *One post-SE animal died during fMRI data collection and was excluded from the study.

Status epilepticus (SE) was induced in rats on a postnatal day (P) 12 (25.6 ± 2.3 g; Fig. 1) as described previously (Kubová et al., 2004). Briefly, all animals received an intraperitoneal (i.p.) dose of LiCl (127 mg/kg) on P11. On P12, the animals were transferred into a silent room and moved into separate cages on a heating pad set at +33 ± 1 °C to maintain body temperature during the maternal separation period (Conklin and Heggeness, 1971). Animals were randomly allocated into SE and control groups. SE was induced by a single i.p. dose of pilocarpine (35 mg/5 ml/kg – 35 mg dissolved in 5 ml of saline; 5 ml/kg injected), while controls were injected with a corresponding amount of saline. Latency to motor SE characterised by forelimb clonus was registered. After 1.5 h of convulsive SE, animals were injected with paraldehyde (0.07 ml/kg) to decrease the mortality rate, followed by subcutaneous injection of 0.5 ml of saline to compensate for the volume loss. After a brief recovery (approximately 15 min), the pups were returned to their dams. Control animals were treated with a corresponding dose of paraldehyde and with the same duration of maternal separation (approximately 4 h). Subsequently, animals were weighed daily for one week and received an injection of 0.5 ml of saline each day when they had not gained weight. More information about SE induction is available in Supplementary material.

### 2.3. MRI

Four animals (two SE and two controls) were used in pilot MRI measurements at P21 and P28 to optimise measurement setup and timepoints for MRI data collection. For further MRI experiments, five litters of animals from different parent pairs were used – four animals per litter with an even number of post-SE rats and controls were selected from the first three litters. Due to the sudden death during the MRI scanning at the first time point (P28), two control animals were excluded from the study and replaced with two additional controls selected from each of the last two litters (5 animals per litter). Animals belonging to the same litter were reared together by the same dam. Structural MRI data were collected from 10 SE and 10 control animals in two time points: P28 – around the peak in myelination rate; and 3 months after SE induction – after the full myelin development when SE animals were in the chronic stage of epileptogenesis (Downes and Mullins, 2014; Nairismagi et al., 2006). Three days after the second MRI measurement, BOLD fMRI data were acquired. One SE animal died during fMRI acquisition, leading to a total of 9 SE and 10 control datasets. Incomplete data were excluded from further analyses.

A horizontal 7 T magnet system (Bruker PharmaScan; Bruker BioSpin MRI GmbH, Ettlingen, Germany) with ParaVision 5.1 software was used for all MRI measurements, including BOLD fMRI. In each measurement, the animal was placed on the MRI rat holder with bite and ear bars for head-fixing and warm-water circulation for maintaining the body temperature at 37 °C. The breathing rate and the body temperature were monitored through the whole procedure with a pneumatic sensor pad and a rectal probe, and adjusted as necessary by changing the isoflurane concentration or warm water flow, respectively.

### 2.4. Structural MRI

Animals were anesthetised with isoflurane (1.5%; O_2_/N_2_ 30/70% as carrier gas) maintaining the breathing rate at 60–70 breaths per minute. Structural T_2_-weighted images were obtained with a fast spin-echo sequence using the following parameters: echo spacing 10.7 ms, effective echo time (TE) 42.7 ms, echo-train length 8, repetition time (TR) 2570 ms, four averages, matrix size 256 × 256, field of view (FOV) 38.4 × 38.4 mm^2^, 24 consecutive slices, and 0.5 mm slice thickness. Quantitative relaxation mapping with RAFF4 along with MT and DTI acquisitions were performed aiming at detecting myelin-related changes. Scanning sessions with a mean duration of 2 h 32 min were conducted.

Diffusion-weighted data were obtained using 3D spin echo-planar imaging (EPI) sequence with 42 uniformly distributed directions with diffusion weighting (b-value 2800 s/mm^2^), TR 1000 ms, TE 26 ms, FOV 24 × 18 × 12.25 mm^3^, encoding matrix 96 × 54 × 49, spatial resolution 0.25 × 0.25 × 0.25 and reconstruction data matrix 96 × 72 × 49. DTI data were corrected for motion and eddy currents using ExploreDTI (Leemans et al., 2009) and further processed using in-house MATLAB scripts ((Mathworks, 2016); release 2017b). The diffusion tensor was determined at each voxel using a nonlinear least-squares algorithm and the eigenvalues (λ_1_, λ_2_, and λ_3_) and eigenvectors (V_1_, V_2_, and V_3_) were calculated. Eigenvalues and eigenvectors were used in the construction of fractional anisotropy (FA), mean diffusivity (MD), and axial and radial diffusivity (AD, RD) maps (Basser et al., 1994; Pajevic and Pierpaoli, 1999).

Tract-based spatial statistics (TBSS) (Smith et al., 2006) was applied to the FA data using the FMRIB Software Library (FSL) (Smith et al., 2004). First, all FA images at respective time points were co-registered to the common FA target. Mean FA images were constructed from the co-registered images and skeletonised (FA threshold >0.3). FSL brain extraction tool (Smith, 2002) was used on B0 map of the corresponding co-registration FA target to create the brain mask used in skeletonisation. A voxel-wise two-sample permutation test, comparing SE and control groups, was applied to the skeletonised FA data using the FSL randomise tool with 10,000 permutations. Family-wise error (FWE) correction was used to account for multiple comparisons (*p* < 0.05).

### 2.5. Relaxation mapping

The imaging slice for the Relaxation Along a Fictitious Field in the rotating frame of rank 4 (RAFF4) and MT scans was selected from the T_2_-weighted images at the position with heterogeneous myelin content, where grey matter structures typically damaged in TLE models (e.g. hippocampus, thalamus, and piriform cortex) are located −3.2 mm from the bregma in the coronal plane based on the brain atlas (Paxinos and Watson, 2017). We applied RAFF4 since it exhibits faster relaxation than RAFF5 while maintaining a high contrast between grey and white matter correlating with myelin content (Hakkarainen et al., 2016). The following parameters of fast spin-echo readout pulse sequence were used to obtain RAFF4 data: TR 4000 ms, TE 8.3 ms, matrix size 256 × 256, FOV 32 × 32 mm, number of echoes 8, number of slices 1, slice thickness 0.5 mm. The duration of a single RAFF4 pulse was 4.525 ms and the peak RF amplitude was γB1 = 323 Hz. The RAFF4 pulses were applied in incremental pulse trains (pulse train durations 0, 108, 217, 326, and 434 ms) to measure signal decay with and without an inversion pulse (hyperbolic secant, duration 8 ms, peak amplitude γB1 = 2500 Hz) to account for steady state formation (Liimatainen et al., 2015; Liimatainen et al., 2010). MT measurements were conducted from the same slice location and using the same readout sequence as for RAFF4. The MT protocol using inversion recovery during off-resonance irradiation was used (Mangia et al., 2011). The signal decay during off-resonance irradiation was acquired with and without an inversion pulse (hyperbolic secant, duration 8 ms, γ*B*_1_ = 2500 Hz) before the off-resonance irradiation. A square saturation pulse (γ*B*_1_ = 200 Hz) with incrementing pulse duration of 0, 0.3, 0.6, 0.9, and 1.2 s was placed 8 kHz off-resonance.

**Fig. 2.**
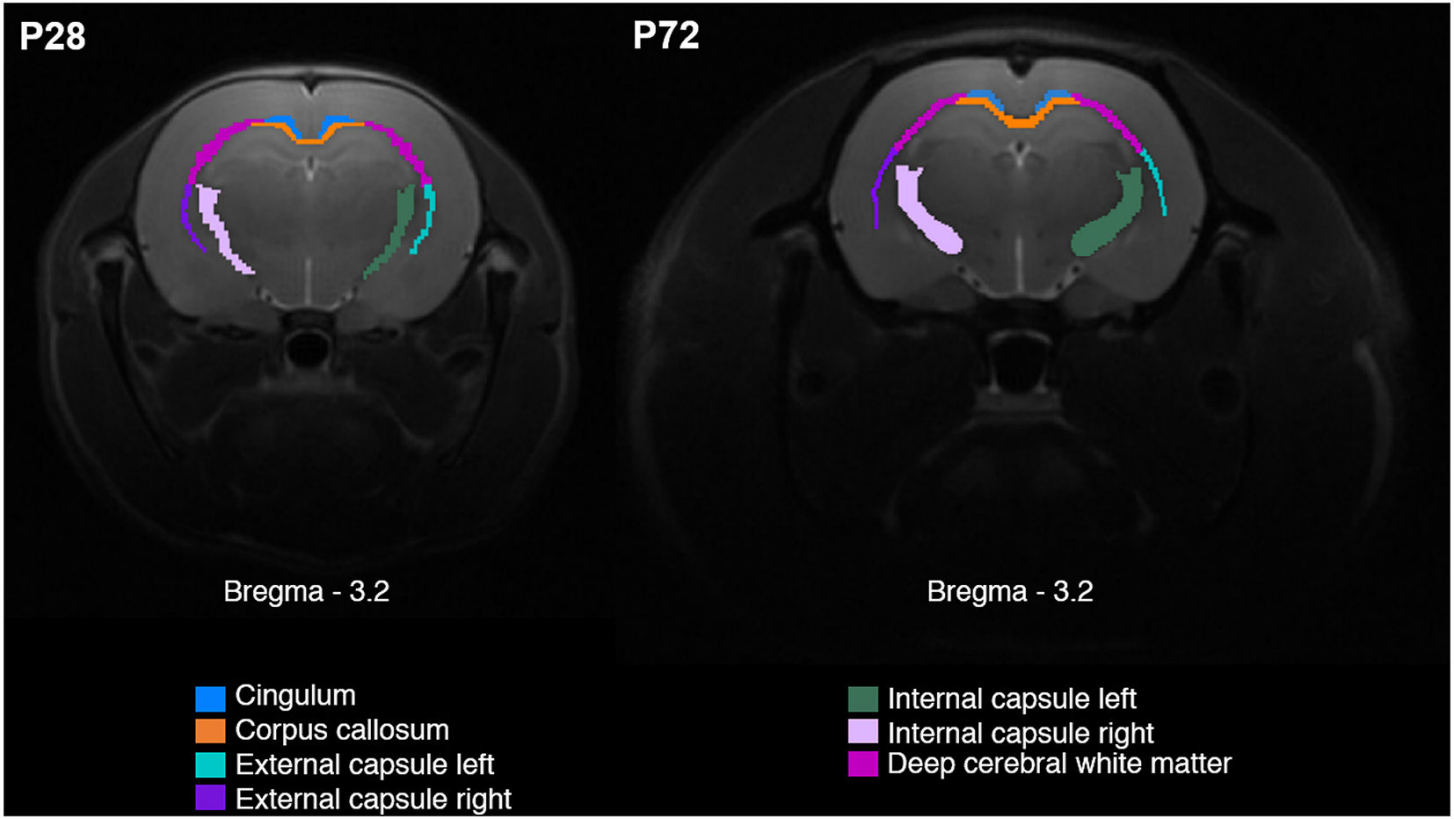
Regions of interest (ROIs) representing white matter bundles. ROIs located at coronal plane −3.2 mm from the bregma used for the analysis of white matter integrity in MRI parametric maps (MTR, T_1sat_, RAFF4, FA, AD, MD, and RD) on postnatal days 28 and 72. ROIs are overlaid on the structural T_2_ relaxation reference brain.

The relaxation time maps were reconstructed from signal intensities on a pixel-by-pixel basis using MATLAB (Mathworks, 2016). T_RAFF4_ values were calculated using monoexponential decay and rise functions to the same steady-state value, as described earlier (Liimatainen et al., 2010). From the MT data, longitudinal relaxation during off-resonance irradiation T_1sat_, steady-state magnetisation M_SS_ and magnetisation in a fully relaxed state, M_0_, were solved similarly as with RAFF4 taking the steady-state into account (Mangia et al., 2011). MT ratio (MTR) was calculated as MTR = 1–M_SS_/M_0_. The regions of interest (ROIs) corresponding to white matter structures were hand-drawn using the Aedes software package (Niskanen, 2006). The list of ROIs included: corpus callosum (CC); cingulum (CIN); internal capsule in left (ICL) and right (ICR) hemisphere; deep cerebral white matter (DCW); and the external capsule in left (ECL) and right (ECR) hemisphere (Fig. 2). All maps were co-registered to reconstructed T_2_-weighted images of the reference brain at individual time points using ANTs software (Avants et al., 2011). ROIs were compared between SE and control animals in relaxation time maps (RAFF4, MTR) and corresponding slice position in FA maps using two-sided Wilcoxon-Mann-Whitney test with false discovery rate correction (FDR) in RStudio (version 1.2.5033 (R Core Team, 2017)).

### 2.6. fMRI

To assess changes in brain connectivity, BOLD fMRI measurements were performed as a final experiment under (terminal) urethane anaesthesia induced with three equal consecutive intraperitoneal doses (in total 1750 mg/kg) over 15 min. During the administration of urethane, a gradually decreasing concentration of inhaled isoflurane was used. After initial shimming, functional images of the whole brain were acquired using gradient-echo EPI (GE-EPI) with parameters: TR 1000 ms, TE 18 ms, matrix 64 × 64, 200 kHz bandwidth, FOV 28.8 × 28.8 mm, flip angle 60°, slice thickness 1 mm, 15 slices, and a total of 3600 acquisition time points. Before the analyses, the resting-state fMRI data were converted to NIfTI format using the Aedes software package (Niskanen, 2006). Standard pre-processing was done, including slice-timing correction, motion correction, and co-registration to the reference brain using ANTs software (Fmriants by stnava, 2021). Data analysis was performed using in-house written MATLAB scripts ((Mathworks, 2016); release R2018b).

For the functional connectivity analysis, ROIs were drawn covering the thalamic and cortical areas (14 ROIs; Fig. 5A) and the default mode network (DMN; 11 ROIs) respectively (Fig. S1). Partial correlations were calculated for each connection between the cortex and thalamus or within the DMN while using motion correction parameters as regressors to compensate for the motion effects. Urethane-anesthetised rats experience transitions between two sleep-like brain states (Clement et al., 2008), and the breathing rate changes in transition between these two states (Pagliardini et al., 2012). Breathing changes are also reflected in the BOLD signal. Therefore, we used a breathing rate aligned with the BOLD signal as an additional regressor for partial correlation calculation. The correlation coefficients were normalised with Fisher’s z-transformation before group-wise testing. The comparison between groups was performed using a permutation test with 10,000 permutations, with false discovery rate (FDR) correction for multiple comparisons ((Mathworks, 2016); release R2018b). The criterion for statistical significance was set at FDR-corrected *p* ≤ 0.05.

### 2.7. Perfusion and histological procedures

Animals that underwent MRI were perfused with 4% paraformaldehyde (PFA) on P75, immediately after the fMRI measurement. An additional group of 50 animals was included in the study (5 litters with pups randomly assigned to both SE and control groups) to address the effect of epileptogenesis on myelination throughout postnatal myelin development using histological staining (Fig. 1). Animals were randomly assigned to SE and control groups, and allocated for tissue collection at postnatal days 15, 18, or 28 (P15, P18, and P28), ensuring that each group contained animals from multiple dams. Pups were fostered after birth. An equal number of SE and control animals per group were allocated for P15 (eight SE and eight controls) and P18 intervals (ten SE and ten controls). More animals were allocated for SE induction for the P28 interval compared with controls (eight SE and six controls) to compensate for potential post-induction mortality. The survival rate was 100% in all three age groups. Animals were sacrificed by urethane overdose (2.5 g/kg, i.p.) and perfused with 4% PFA at P15 (*n* = 16), P18 (*n* = 20) and P28 (*n* = 14). Whole brains, including the cerebellum and olfactory bulb, were collected from all animals and post-fixed for 3–4 h in 4% PFA. In order to successfully perform staining to assess the myelin content, the post-fixation period in PFA was prolonged to 36 h. The brains were subsequently incubated in cryoprotective solution (20% glycerol in 0.02 phosphate-buffered saline) for 36 h, rapidly deep-frozen in dry ice, and stored at −70 °C. The brains were cryo-sectioned in the coronal plane, using a Leica CM1900 Research Cryostat, into 50-μm-thick slices (1-in-5 series). One series of sections was used for the staining of myelin structures by Black-Gold II (Schmued et al., 2008). The sections were viewed under bright-field illumination with an Olympus AX70 microscope equipped with an Olympus DP70 digital camera and Olympus Micro Image Analysis Software (Media Cybernetics, L.P., Newburyport, MA, U.S.A.).

The optical density (OD) of large white matter structures was quantified across eight Black-Gold II-stained brain sections per animal using ImageJ software (version 1.52 s (ImageJ, 2020)). Analysed sections ranged from −0.7 to −6.2 mm distance from the bregma in adult animals, while corresponding locations were selected in younger animals with respect to the smaller brain size (Fig. S2). OD values were averaged over three ROIs (Fig. S3) in each hemisphere per structure in corresponding brain sections: corpus callosum (bregma −0.7, −1.7, −2.2, −3.2, −3.7 and − 4.7 mm); forceps major of corpus callosum (bregma −5.7 mm); deep cerebral white matter (bregma −4.7, −5.7 and −6.2 mm) and internal capsule (bregma −0.7, −1.7, −2.2, −3.2, and −3.7 mm). The reference background value was obtained along with ROI collection and subtracted from OD values of individual ROIs. Prior to statistical evaluation, the normality of the data (both raw and transformed to logarithmic scale) distribution was tested and rejected by the Shapiro-Wilk test using RStudio (version 1.2.5033 (R Core Team, 2017)). Wilcoxon-Mann-Whitney test was used to compare the optical density of individual white matter structures between post-SE animals and controls in each age group.

## 3. Results

### 3.1. SE induction and MRI pilot

Status epilepticus (score 3) was induced successfully in all animals injected with pilocarpine with mean latency of 925 ± 347 s (min = 510 s and max 2225 s) and 100% post-induction survival rate during and after SE. One day after SE, rats with SE lost weight whereas controls gained bodyweight (−1.8% ± 6.4% vs. + 14.5 ± 2.8%; *p* < 0.0001) as revealed by Mixed-effects analysis (F_(15,600)_ = 23.556; p < 0.0001). Thereafter, the day-by-day analysis revealed no differences and both SE and control animals gained 3% to 20% of body weight daily (Fig. S4). During the MRI pilot study, we scanned animals at P28 and P21. We observed inconsistent breathing rates at P21 under isoflurane anaesthesia (oscillating from 10 to 100). The inconsistency dissipated at the age of P28. Hence, P28 was selected as the early time point for further MRI scanning.

### 3.2. Altered myelination following SE

**Fig. 3.**
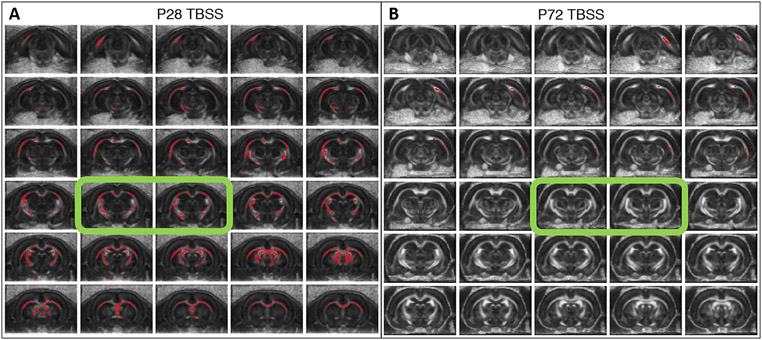
Difference in myelin integrity between post-SE animals and controls – TBSS analysis applied on DTI data shows an increase in fractional anisotropy (FA) value in rats with epileptic status (SE) on 12^th^ postnatal day (P12) compared with controls (FDR corrected *p*-value ≤0.05 indicated by red colour). First DTI measurement performed on P28 (**A**) detected increased FA in major white matter structures (corpus callosum, fornix, deep cerebral white matter, and internal capsule) across the whole detected space. The displayed area corresponds approximately to range from −7.50 to +0.30 distance from the bregma in the adult animal. The same group of animals shows only a discrete increase in FA values in deep cerebral white matter 3 months after SE (**B**); displayed range: Bregma −7.50 to −0.25. Green rectangles indicate the position corresponding to RAFF4, MTR and T_1sat_ measurements.

**Fig. 4.**
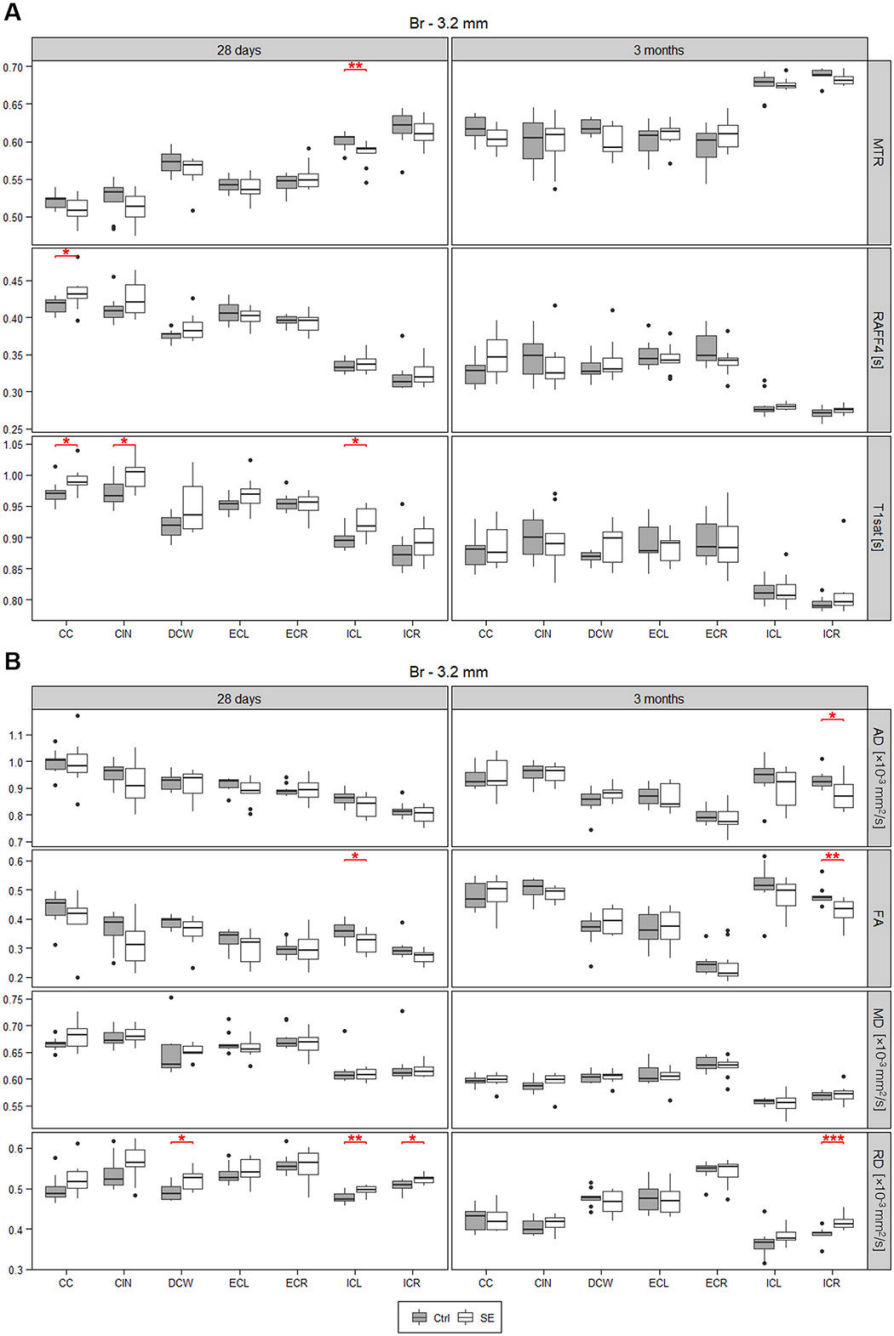
MRI outcomes in primary white matter ROIs. MRI maps addressed white matter integrity in animals with epileptic status (SE) on a postnatal day 12 (P12) and controls (Ctrl) within two consecutive measurements on P28 and P72. Relaxation maps (**A**: MTR = Magnetisation transfer ratio, T_1sat_ = T1 relaxation time under magnetisation transfer irradiation, and RAFF4 = Relaxation Along a Fictitious Field), and DTI (**B**: FA = fractional anisotropy; AD = axial diffusivity, MD = mean diffusivity and RD = radial diffusivity) data were collected on a single coronal 0.5 mm thick slice at a position −3.2 mm from the bregma. White matter boundless (CC = corpus callosum; CIN = cingulum; DCW = deep cerebral white matter; ECL, ECR = external capsule left, right; ICL, ICR = internal capsule left, right) were compared using Wilcoxon-Mann-Whitney test (* *p* ≤ 0.05; ** *p* ≤ 0.01; *** *p* ≤ 0.001 and **** *p* ≤ 0.0001, red).

TBSS analysis of DTI data showed widespread changes in white matter occurring at P28 in animals after SE. An increase in FA values was detected in all large white matter bundles in both hemispheres (corpus callosum, cingulum, internal capsule, external capsule, etc.) ranging from −0.2 to −7.50 mm from the bregma in the coronal plane (Fig. 3), indicative of compromised white matter integrity. ROI analysis confirmed this trend with up to a 12% decrease in FA between SE animals and controls, accompanied by increased (~4%) radial diffusivity (RD) (Fig. 2, Table S1) consistent with decreased myelination or myelin damage. At 3 months, the difference between SE and control animals was present only in the external capsule and the deep cerebral white matter across the range from −4.0 to −6.75 mm from the bregma in the left hemisphere in FA TBSS analysis (Fig. 3) and in the external capsule in ROI analysis. In this case, a FA decrease of ~12% was associated with ~8% RD increase and ~6% axial diffusivity decrease consistent with myelin damage and axial damage, respectively. ROI analyses of the relaxation maps at a position −3.2 mm from the bregma in the coronal plane showed an increase in RAFF4 relaxation rate and T_1sat_ relaxation time constants of the corpus callosum in SE animals on P28. The increase in RAFF4 and T_1sat_ relaxation rate is consistent with a decreased amount of myelin. T_1sat_ values were also increased in the post-SE cingulum and left internal capsule on P28. Comparably, MTR showed decreased values in the left internal capsule of SE animals (Fig. 4) at the early time-point. Similar to TBSS results, these alterations were no longer detectable by any of these methods three months after SE (Fig. 4, Table S1).

### 3.3. SE alters myelin development across multiple postnatal stages

**Fig. 5.**
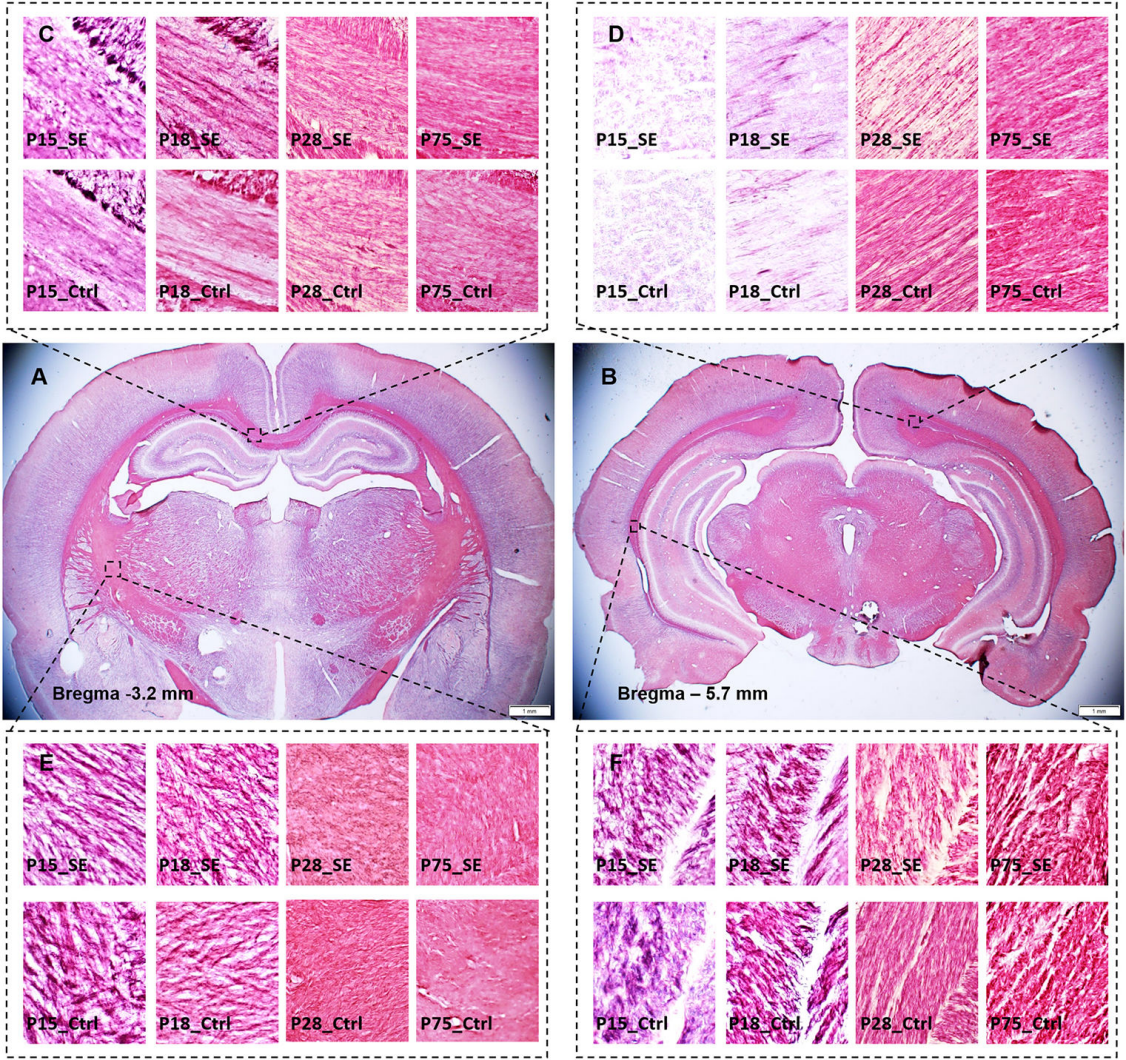
White matter structures across stages of epileptogenesis -representative Black-Gold II staining on brain sections located approximately −3.2 (**A**) and −5.7 (**B**) mm from the bregma. The middle row shows an overview of the brain sections in an adult animal. Black squares indicate the location of the close-ups representing corpus callosum (**C**), internal capsule (**D**), forceps major of corpus callosum (**E**), and deep cerebral white matter (**F**). The upper row of close-ups corresponds to animals with status epilepticus (SE) induced at postnatal day 12 (P12), lower displays control (Ctrl) tissues across four tested ages (P15, P18, P28, and P75) respectively.

**Fig. 6.**
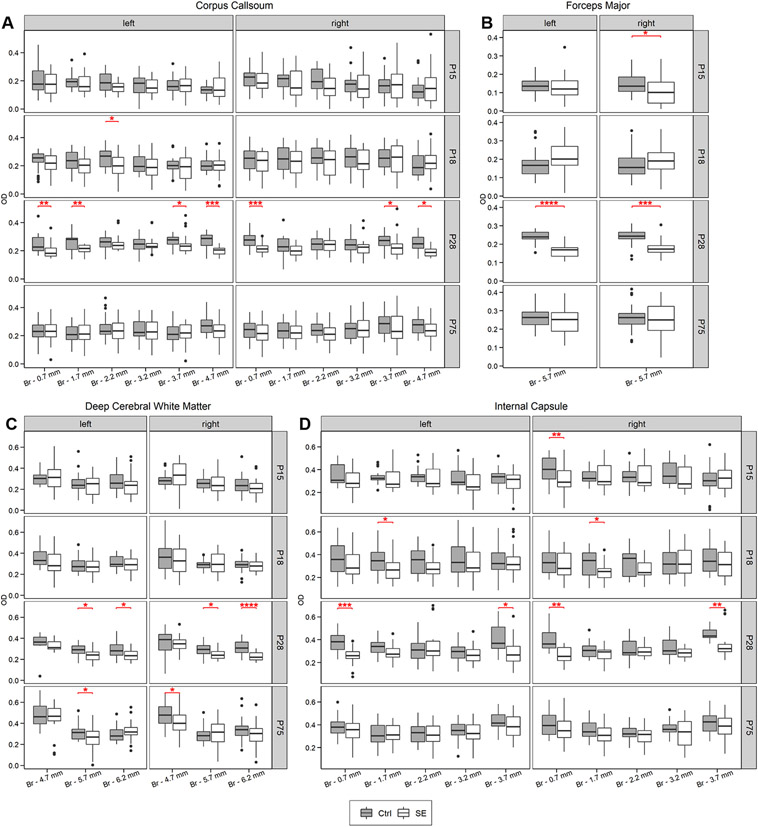
Myelin content evaluation in white matter structures across epileptogenesis. Results of optical density analysis of corpus callosum in right and left (**A**) hemisphere, forceps major of the corpus callosum in both hemispheres (**B**), deep cerebral white matter (**C**), and internal capsule in left and right hemisphere (**D**) in 15, 18, 28 and 75 days old rats. White boxes represent animals with status epilepticus (SE) induced at postnatal day 12 (P12), grey boxes represent age-matched controls. The position of brain sections is displayed as the distance from the bregma (Br) in millimetres. Significant results of the Wilcoxon-Mann-Whitney test are displayed as red stars and brackets in the upper middle part (* p ≤ 0.05; ** p ≤ 0.01; *** p ≤ 0.001 and **** p ≤ 0.0001; red).

Histological evaluation of myelin content in the samples stained by Black-Gold II (Fig. 5) showed age-dependent changes in the myelin integrity across epileptogenesis duration from the early stages following SE (P15, P18) until adulthood (P75) (Fig. 6). On day P15 (3 days after SE induction), we detected significantly decreased optical density in the forceps major of the corpus callosum and the internal capsule of SE rats. However, these alterations were located only at a single position per structure and unilaterally in the right hemisphere. On day P18, the myelin staining density decreased in the SE animals below the values of the control rats bilaterally in the anterior internal capsule and unilaterally in the corpus callosum. The forceps major of the corpus callosum and deep cerebral white matter remained within the normal values observed in controls. Consistent with the MRI results, the myelin content reduced dramatically in SE animals on day P28, where controls show higher myelin density across all tested white matter structures (Fig. 6). These differences dissipated almost entirely in 75-day-old animals (3 months after SE induction), with decreased OD values restricted to deep cerebral white matter in both the right (position − 5.7 mm from the bregma) and left (position − 4.7 mm from the bregma) hemispheres (Fig. 6, Table S2).

### 3.4. fMRI

**Fig. 7.**
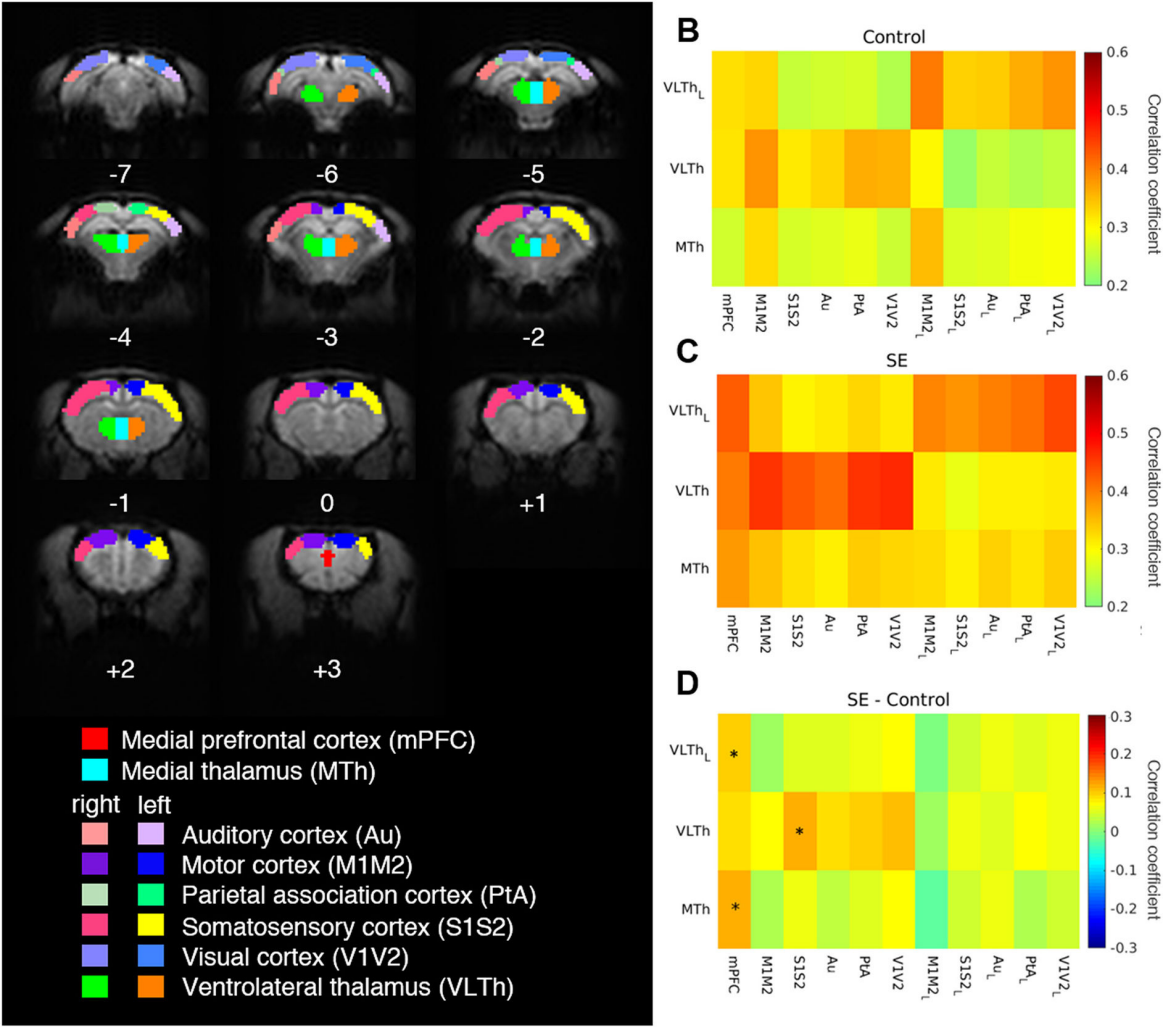
Altered thalamocortical connectivity three months after epileptic status induction. Thalamocortical connectivity was assessed across 14 regions of interest on a postnatal day 75 (**A**). Numbers located under individual brain slices indicate their distance from the bregma in mm. Average group correlation matrices for thalamocortical regions are displayed for control (**B**) and post SE (**C**) rats. Subscript letter L marks regions located in the left hemisphere. The difference between post-SE and control correlation matrices is displayed (**D**) with positive values meaning higher connectivity in the SE group and negative values meaning higher connectivity in the control group (* p ≤ 0.05, FDR-corrected).

fMRI data were obtained from animals at a single time point on P75 (three days after the second MRI measurement was recorded) under urethane anaesthesia. We detected no significant differences in default mode network connectivity. However, thalamocortical connectivity increased in the rats 3 months after SE compared with controls (Fig. 7), with the connection from the medial and left ventrolateral thalamus to the medial prefrontal cortex (mPFC) significantly increased (FDR-corrected *p* = 0.00005; and 0.011, respectively). Similarly, fMRI detected an increase in activity correlation between the right ventrolateral thalamus to the right somatosensory cortex (S1S2) in animals 3 months after SE (FDR-corrected *p* = 0.045).

## 4. Discussion

Well-developed white matter serves a crucial role in signal conduction and timing of the neuronal firing, while myelin loss may change excitability and induce axonal vulnerability (reviewed in (Bercury and Macklin, 2015; de Curtis et al., 2021; Seidl, 2014)). In this study, we addressed myelin integrity in the animal model of early-onset TLE and its potential impact on functional connectivity. Our results show that Lipilocarpine-induced SE in the infantile (P12) rat brain reduces white matter integrity during brain development. White matter integrity was impaired minimally in the early post-SE timepoints (P15 and P18). On the other hand, we observed a widespread reduction of white matter integrity on P28 expanding across major white matter bundles (corpus callosum, deep cerebral white matter, internal capsule, etc.) in both MRI (TBSS applied on FA maps) and myelin-specific histology. These white matter alterations dissipated almost entirely in adult animals (P72) while remaining detectable only in limited areas in the deep cerebral white matter. The subsequent fMRI showed an increase in thalamocortical connectivity from the medial thalamus to the medial prefrontal cortex in adult epileptic animals.

We addressed white matter integrity using a combination of MRI techniques sensitive to white matter disruption in a single slice positioned −3.2 mm from the bregma. FA, MTR, and RAFF4 detected compromised integrity of white matter in SE animals within a single analysed ROI only, while TBSS and T_1sat_ showed larger disruption of white matter at this position on P28. This indicates higher sensitivity of the TBSS approach, as both TBSS and ROI-based FA results were constructed using the same FA maps (Smith et al., 2006). In the single-slice approach, MTR and FA decreased significantly in the left internal capsule on P28 but failed to detect alterations in other ROIs including the corpus callosum, marked as significantly altered by the increase in RAFF4 and T_1sat_. These differences most likely originate from the sensitivity to different white matter characteristics of individual detection methods – FA addresses predominantly microstructural integrity (reflecting features like axonal diameter, myelin thickness, axonal damage, etc.); RAFF4 and MT indicate decreased myelin content in post-SE animals (Holikova et al., 2021; Lehto et al., 2017; Mori and Zhang, 2006). In accordance with other studies comparing white matter sensitive techniques (Lehto et al., 2017), this suggests that rather than being superior to one another, these methods complement each other in determining white matter integrity.

MRI is an invaluable tool in both diagnostics and research due to its noninvasive character, allowing repeated measurements. However, histological analyses provide better resolution, sensitivity, and specificity; hence, they are an irreplaceable validation technique for MRI findings in animal models. The limitation of this study is the lack of MRI data for early post-SE timepoints (P15 and P18), which were not obtained due to the breathing inconsistencies during anaesthesia observed in pilot MRI measurements. The animals examined by MRI on P28 were measured again on P72 to address longitudinal changes after early myelination defects with fMRI follow-up on P75. Hence, two different groups were analysed on P28 by MRI and histology, respectively, explaining the minor discrepancies between detected white matter alterations observed by individual techniques. Nevertheless, the results of both techniques show altered white matter integrity across all tested bundles (internal capsule, corpus callosum, deep cerebral white matter). Three months after SE induction, the histology results on P75 are mostly consistent with the MRI (TBSS) data on P72 showing homologous values in the post-SE animals and controls. The only discrepancy between the two techniques in the measurement of adult animals lies within the decreased post-SE values detected (unilaterally, and bilaterally) in the deep cerebral matter (Fig. 2 vs Fig. 5).

Increased RD points towards myelin damage or reduced myelin thickness rather than axonal damage as a predominant mechanism behind detected MRI changes (Song et al., 2005), even though this classical interpretation has been recently challenged (Lehto et al., 2017). In the present study, this interpretation is supported by the recovery of the white matter in adulthood. In one brain area, compromised white matter integrity was detected still in adulthood; in this case, both increased RD and decreased AD were detected, implying that early myelin damage can trigger axonal damage in some cases. In our model, we observed the first white matter abnormalities with a slight delay from TLE onset, while the most prominent disruption followed the peak of myelination. This might be explained by the typical fast increase in myelin content and white matter density at this age (around P24 for the corpus callosum and capsules) (Downes and Mullins, 2014), leading to white matter development impairment observed on P28. After this period, the white matter volume and myelin content increase more gradually, and further development along with adaptive myelination (Bercury and Macklin, 2015; Downes and Mullins, 2014) recovers this disparity between the epileptic and control brains. To understand this process, further investigation of white matter integrity between postnatal days 28 and 72 in the early-onset epilepsy models and involvement of electron microscopy would be beneficial in future studies.

Proper development of myelin is essential for the formation of fast and effective neuronal connections. In a healthy brain, neuronal activity leads to an increase in myelination, supporting the transduction of signals within a neuronal network. In the case of epilepsy, aberrant neuronal activity during seizures may inhibit myelin formation despite the normal or increased presence of oligodendrocyte precursor cells (Gibson et al., 2018; Zucchini et al., 2014). In epileptic patients, myelin alterations are frequently reported. Nevertheless, their occurrence is reported inconsistently across variable areas. The most common myelin changes in adult TLE patients occur in the temporal lobe white matter, frontotemporal connections, cingulum, and corpus callosum ipsilateral to the epilepsy foci with a frequent bilateral spread of the white matter reduction (Arfanakis et al., 2002; Concha et al., 2009; Liu et al., 2016; Schoene-Bake et al., 2009; Urbach et al., 2017). Furthermore, the white matter abnormalities occur frequently in early-onset TLE with hippocampal sclerosis, suggesting its impact on myelin development (Garbelli et al., 2012). Studies focused on children with TLE report white matter reduction even less consistently, with temporal lobe white matter being the only repeatedly affected region (Bartolini et al., 2017; Govindan et al., 2008; Kimiwada et al., 2006; Nilsson et al., 2008). Nevertheless, these studies address the white matter in a wide age range from birth until adulthood without considering the effect of brain development on these changes. As in patients, myelin damage has been reported across the spectrum of animal models of epilepsy (Salo et al., 2017; Sierra et al., 2011; van Eijsden et al., 2011). In Li-pilocarpine epilepsy induced in adult rats, the myelin disruption was observed in the hippocampal area (Luo et al., 2015; Ye et al., 2013). Similar to our data, Eijsden and colleagues detected recovery of corpus callosum white matter between the fourth and eighth week after SE in juvenile (P21) rats (van Eijsden et al., 2011). Fornix fimbriae damage persisted in these animals until adulthood. We addressed white matter changes in Li-pilocarpine-induced epilepsy in the infantile brain and we observed a similar pattern of the white matter disruption and recovery in multiple myelinated structures.

Even though the white matter development catches up in adulthood, the epileptogenesis induced in the infantile brain evolves further, eventually manifesting in seizures and brain damage (Kubová and Mareš, 2013; Nairismagi et al., 2006). At P12 the rat brain undergoes maturation processes beyond white matter development (e.g. network formation, rapid synaptogenesis and hippocampal neurogenesis), hence the consequences of SE at this age differ from those observed in more mature animals. The developing brain appears less vulnerable to neuronal loss while maintaining brain damage in structures such as the hippocampus, mediodorsal thalamic nuclei, or piriform cortex (Kubová and Mareš, 2013; Nairismagi et al., 2006). The development of motor functions is initially slowed down and similarly to the white matter disruption, the motor impairment is transient and recovers later in life (Kubová and Mareš, 2013). Animals with TLE onset in the immature brain exhibit milder cognitive and behavioural impairment than their adult-onset counterparts. However, their memory, learning, social behaviour, and anxiety levels are still deviant from control populations (Kubová and Mareš, 2013; Mikulecká et al., 2019; Nairismagi et al., 2006; Rutten et al., 2002). Starting at P25, animals exposed to SE at P12 exhibit increased anxiety followed by deficits in sociability and social discrimination in adulthood (Mikulecká et al., 2019). Impaired functional connectivity arising from altered myelin development might be involved in these cognitive and behavioural malformations. Here we addressed the functional brain connectivity of adult animals with SE in the developing brain. Although connectivity in the default mode network is frequently reported as altered in epileptic patients as well as animal models with epilepsy induced in adulthood (Centeno and Carmichael, 2014; Cui et al., 2018; Gill et al., 2017; Grassia et al., 2018; Haneef et al., 2014), our analysis did not show any significant change in the connectivity of these regions. The reason for this discrepancy might be the low number of subjects in the fMRI analysis, which involved only seven SE animals and seven controls due to the presence of the artefacts from the urethane sleep-like state transitions and motion-related artefacts that we were not able to subtract from the fMRI signal of the remaining animals. On the other hand, the number of animals was sufficient for the detection of increased corticothalamic connectivity from the medial and left ventrolateral thalamus to the medial prefrontal cortex and right ventrolateral thalamus to the right somatosensory cortex in post SE animals. Due to its rich connections to cortical structures, the thalamus poses as a relay center for seizures and is involved in seizure initiation and propagation (Haneef et al., 2014; Holmes et al., 2013; Li et al., 2014; Paz et al., 2013). Similarly to humans, the rat mPFC is important for learning; hence its altered connectivity may provide (along with the hippocampal pathology typical for this model) a partial explanation for impaired learning observed in behavioural studies (Kubová and Mareš, 2013; Letty et al., 1995; Mikulecká et al., 2019; Narayanan et al., 2013). However, there is some controversy regarding the level of overlap between the human and rodent prefrontal cortex, both on the anatomical and functional level; these functional implications need to be taken with caution and might not be entirely relatable to changes detected in humans (Laubach et al., 2018).

## 5. Conclusion

In conclusion, we have addressed the integrity of large white matter structures in the epileptic brain across the period of postnatal myelin development until adulthood. Our results show that early-onset TLE has a distinct impact on white matter integrity in the developing brain. The extent of white matter reduction correlates with myelin development in the rodent brain. The majority of the white matter disruption recovers in adult animals, suggesting delayed development as a plausible source of the observed changes in the post-SE brain. Despite this recovery, *epi*lepsy progresses further, resulting in increased thalamocortical connectivity. Our results indicate that addressing white matter integrity during the critical period of myelin development in early-onset TLE is a worthwhile endeavour that might prove useful in TLE patient cohorts and stresses the importance of the *epi*lepsy onset age.

## Supplementary Material

1

## Abbreviation list:

AD: axial diffusivity
ADHD: attention deficit hyperactivity disorder
BOLD: blood oxygenation level dependent
CC: corpus callosum
CIN: cingulum
DCW: deep cerebral white matter
DMN: default mode network
DTI: diffusion tensor imaging
ECL / R: external capsule left / right
EPI: echo-planar imaging
FA: fractional anisotropy
FDR: false discovery rate
fMRI: functional magnetic resonance imaging
FOV: field of view
FSL: FMRIB Software Library
FWE: family-wise error
ICL / R: internal capsule left / right
MD: mean diffusivity
mPFC: medial prefrontal cortex
MT: magnetisation transfer
MTR: magnetisation transfer ratio
OD: optical density
P: postnatal day
RAFFn: Relaxation Along a Fictitious Field in the rotating frame of rank n
RD: radial diffusivity
ROIs: regions of interest
S1S2: somatosensory cortex
SE: status epilepticus
TBSS: Tract-based spatial statistics
TE: echo time
TLE: Temporal lobe epilepsy
TR: repetition time
