# Supplementary material for: Infantile status epilepticus disrupts myelin development
Source: Neurobiol Dis. Author manuscript; Available in PMC 2022 Feb 15. (PMC8845085; doi:10.1016/j.nbd.2021.105566)
Supplement: 1 [file NIHMS1775908-supplement-1.pdf]

Supplementary methods

On postnatal day (P) 11, the animals were marked for identification and randomly assigned to SE or control groups, ensuring that each group contained animals from multiple litters to exclude a litter effect. Each litter consisted of both controls and SE exposed animals. In the group of additional animals for histology, the rats were at the same time randomly allocated for tissue collection at postnatal days 15, 18, or 28 (P15, P18, and P28) while controlling for the litter effect. All pups received LiCl (127 mg/kg; LiCl 127 mg was dissolved in 5 ml of water for injection and injected intraperitoneally 127 mg/5ml/kg) and were returned to their dams. On P12 pups were separated from their dams and transferred into a special silent room with controlled conditions. Experiments were always performed at the same time of the day, between 10 am and 2 pm (i.e. during the light period). Animals were placed individually into small containers and they were maintained at +33±1°C to compensate for the immature thermoregulation at this age (Conklin and Heggeness, 1971) during the entire period of separation from their mothers. SE was induced with a single intraperitoneal injection of pilocarpine (35mg/kg; 35 mg of pilocarpine was dissolved in 5 ml of saline and injected). Controls received corresponding volume of saline instead.

The severity of motor SE was assessed using the following scoring system:

0 – normal behaviour

1 – stereotypic behaviour (face washing, scratching), isolated myoclonic jerks

2 –head bobbing, pivoting, swimming movements

3 – clonic seizures with preserved righting reflexes

4 – repeated periods of wild running

5 – generalized tonic-clonic seizures with loss of righting reflexes.

Animals were assigned a score for the most severe behavioural characteristics. Latency to the onset of motor seizures was recorded. Mortality was recorded throughout the entire experimental period. Only rats that exhibited behavioural manifestations of seizures progressing to forelimbs clonus (i.e., score 3) for at least 1 hour and without periods of wild running and generalized tonic-clonic seizures (score 4-5) were used for further experiments.

After 1.5 hours of convulsive SE, animals were given a single dose of paraldehyde (0.07ml/kg; 0.07 ml of paraldehyde was mixed with 5 ml of saline and injected intraperitoneally). Controls received paraldehyde in the same dose. Approximately 30 min later they were injected subcutaneously with 0.5 ml saline to restore the volume loss. After the brief recovery, pups were returned to their dams (the duration of isolation from mothers in the control and SE groups was the same ~ 4 hours).

The weight of pups was checked daily and animals that did not gain any weight within 24hrs after SE were given 0.5ml of saline with 10% of glucose subcutaneously to prevent dehydration and further weight loss. To minimize the effects of variability in individual groups, the data were used to calculate relative body weight (body weight at P11 was taken as 100%). The difference in the relative body weights between two consecutive days (starting at P11) was used as a measure of weight gain (Figure S4). Statistical comparison of relative weight gains between controls and SE animals was done using RStudio (version 1.2.5033). Data were compared using Mixed-effects analysis with False Discovery Rate (FDR) correction for multiple comparisons. Mixed effect analysis was used instead of One-way repeated measure ANOVA because of missing values.

Statistical disclaimer:

Multiple testing correction techniques are known to reduce false-positive discovery while increasing the risk of type II error (false-negative results)(Drachman, 2012; Ranganathan et al., 2016; White et al., 2019). To avoid the possible unnecessary introduction of false-negative values in cases when the likelihood of false-positive discovery is below 1 per compared set, we consider p-value without adjustment (displayed in table S1 and S2) a true p-value in our ROI statistics with less than 20 comparisons per method (S1) or position (S2).

Table S1 Relaxation map value comparison between post-SE rats and controls

structure (ROI)	MRI modality	Postnatal day 28			Postnatal day 72		
		difference	p	p-adj	difference	p	p-adj
Cingulum	AD	-3.5%	0.248	0.693	-0.1%	0.912	0.912
	FA	-12.2%	0.218	0.433	-2.7%	0.315	0.471
	MD	0.9%	0.280	0.787	1.3%	0.105	0.787
	MTR	-2.4%	0.190	0.444	0.3%	0.739	0.739
	T1sat	2.0%	**0.019**	0.108	0.9%	0.838	0.971
	RAFF	3.5%	0.315	0.505	3.9%	0.218	0.433
	RD	4.7%	0.190	0.333	2.9%	0.190	0.333
Corpus Callsoum	AD	-0.4%	0.684	0.912	1.0%	0.739	0.912
	FA	-7.8%	0.248	0.433	1.7%	0.529	0.617
	MD	2.2%	0.166	0.787	0.3%	0.481	0.971
	MTR	-2.3%	0.105	0.294	2.5%	0.075	0.263
	T1sat	3.0%	**0.023**	0.108	-0.3%	0.661	0.971
	RAFF	3.9%	**0.029**	0.403	-6.5%	0.089	0.433
	RD	5.4%	0.063	0.176	-0.6%	0.912	0.912
Deep Cerebral White Matter	AD	-1.3%	0.867	0.912	3.2%	0.236	0.693
	FA	-9.1%	0.232	0.471	9.5%	0.370	0.471
	MD	0.6%	0.281	0.787	0.2%	0.743	0.971
	MTR	-2.3%	0.336	0.549	3.2%	0.059	0.263
	T1sat	3.7%	0.232	0.464	2.4%	0.230	0.464
	RAFF	2.9%	0.397	0.505	-3.9%	0.370	0.505
	RD	5.8%	**0.040**	0.140	-2.6%	0.370	0.576
External Capsule Left	AD	-2.8%	0.165	0.693	-0.3%	0.912	0.912
	FA	-7.9%	0.353	0.471	1.6%	0.912	0.912
	MD	-1.5%	0.190	0.787	-0.9%	0.796	0.971
	MTR	-1.0%	0.481	0.561	-1.5%	0.436	0.561
	T1sat	1.7%	0.089	0.312	-0.9%	0.713	0.971
	RAFF	-1.5%	0.393	0.505	1.4%	0.796	0.853
	RD	1.5%	0.481	0.673	-1.4%	0.631	0.736
External Capsule Right	AD	0.3%	0.853	0.912	-1.3%	0.436	0.872
	FA	-1.3%	0.739	0.796	-2.3%	0.280	0.471
	MD	-1.1%	0.971	0.971	-0.9%	0.739	0.971
	MTR	1.7%	0.481	0.561	-2.4%	0.353	0.549
	T1sat	-0.3%	0.971	0.971	-0.5%	0.838	0.971
	RAFF	-0.9%	0.684	0.737	4.6%	0.218	0.433
	RD	-0.4%	0.796	0.857	-0.6%	0.529	0.673
Internal Capsule Left	AD	-3.3%	0.165	0.693	-4.6%	0.315	0.735
	FA	-10.6%	**0.043**	0.294	-7.2%	0.218	0.471
	MD	-1.0%	0.912	0.971	-0.3%	0.796	0.971
	MTR	-2.7%	**0.009**	0.125	-0.2%	0.529	0.570
	T1sat	2.9%	**0.015**	0.108	0.3%	0.902	0.971
	RAFF	1.1%	0.579	0.675	0.5%	0.247	0.433
	RD	3.8%	**0.007**	**0.048**	5.3%	0.089	0.208
Internal Capsule Right	AD	-1.9%	0.579	0.912	-5.7%	**0.035**	0.496
	FA	-9.6%	0.063	0.294	-11.6%	**0.002**	**0.029**

MD	-1.2%	0.853	0.971	0.4%	0.853	0.971
MTR	-1.1%	0.353	0.549	0.9%	0.075	0.263
T1sat	1.7%	0.190	0.464	2.3%	0.270	0.473
RAFF	1.9%	0.280	0.505	-1.3%	0.247	0.433
RD	3.1%	**0.019**	0.087	7.7%	**0.000**	**0.007**

difference = Relative difference: (SA-Ctrl)/Ctrl)*100; p = Wilcoxon-Mann-Whitney p-value ; p-adj = FDR corrected p

Table S2 Black Gold II optical density comparison between post-SE rats and controls

Structure	ΔBregma [mm]	Hemisphere	Postnatal day 15		Postnatal day 18		Postnatal day 28		Postnatal day 75	
			p	p-adj	p	p-adj	p	p-adj	p	p-adj
Corpus callosum	-0.7	left	0.387	0.522	0.220	0.440	**0.008**	**0.024**	0.734	0.734
Corpus callosum	-0.7	right	0.599	0.685	0.695	0.734	**0.001**	**0.006**	0.388	0.522
Corpus callosum	-1.7	left	0.252	0.383	0.085	0.206	**0.008**	0.084	0.893	0.943
Corpus callosum	-1.7	right	0.292	0.383	0.444	0.508	0.103	0.206	0.311	0.383
Corpus callosum	-2.2	left	0.092	0.295	**0.024**	0.295	0.266	0.447	0.570	0.760
Corpus callosum	-2.2	right	0.072	0.295	0.158	0.335	0.945	0.945	0.168	0.335
Corpus callosum	-3.2	left	0.699	0.815	0.395	0.677	0.423	0.677	0.764	0.815
Corpus callosum	-3.2	right	0.390	0.677	0.511	0.743	0.292	0.677	0.762	0.815
Corpus callosum	-3.7	left	0.856	0.909	0.701	0.909	**0.042**	0.167	0.822	0.909
Corpus callosum	-3.7	right	0.882	0.909	0.909	0.909	**0.014**	0.100	0.224	0.496
Corpus callosum	-4.7	left	0.640	0.793	0.807	0.807	**0.000**	**0.005**	0.071	0.249
Corpus callosum	-4.7	right	0.644	0.793	0.284	0.505	**0.011**	0.091	0.136	0.292
Forceps major	-5.7	left	0.536	0.780	0.059	0.136	**0.000**	0.069	0.384	0.094
Forceps major	-5.7	right	**0.035**	0.111	0.170	0.340	**0.000**	0.123	0.917	0.606
Deep cerebral white matter	-4.7	left	0.797	0.807	0.093	0.249	0.084	0.249	0.804	0.807
Deep cerebral white matter	-4.7	right	0.146	0.292	0.602	0.793	0.549	0.793	**0.020**	0.109
Deep cerebral white matter	-5.7	left	0.932	0.932	0.880	0.932	**0.013**	**0.000**	**0.024**	0.614
Deep cerebral white matter	-5.7	right	0.694	0.925	0.803	0.932	**0.046**	**0.003**	0.341	0.932
Deep cerebral white matter	-6.2	left	0.180	0.287	0.464	0.531	**0.022**	0.087	0.164	0.287
Deep cerebral white matter	-6.2	right	0.251	0.334	0.591	0.591	**0.000**	**0.001**	0.117	0.287
Internal capsule	-0.7	left	0.070	0.186	0.464	0.571	**0.000**	**0.006**	0.392	0.522
Internal capsule	-0.7	right	**0.005**	**0.020**	0.363	0.522	**0.001**	**0.006**	0.138	0.315
Internal capsule	-1.7	left	0.101	0.206	**0.017**	0.090	0.074	0.206	0.943	0.943
Internal capsule	-1.7	right	0.208	0.371	**0.011**	0.084	0.280	0.383	0.085	0.206
Internal capsule	-2.2	left	0.072	0.295	0.149	0.335	0.626	0.771	0.748	0.798
Internal capsule	-2.2	right	0.477	0.694	0.087	0.295	0.687	0.786	0.279	0.447
Internal capsule	-3.2	left	0.103	0.677	0.383	0.677	0.662	0.815	0.249	0.677
Internal capsule	-3.2	right	0.078	0.677	0.947	0.947	0.255	0.677	0.303	0.677
Internal capsule	-3.7	left	0.248	0.496	0.549	0.879	**0.019**	0.100	0.101	0.324
Internal capsule	-3.7	right	0.635	0.909	0.243	0.496	**0.002**	**0.036**	0.299	0.532

ΔBregma – distance from bregma in coronal plane [mm]; p = Wilcoxon-Mann-Whitney p-value ; p-adj = FDR corrected p

Figure S1 – ROIs representing the default mode network. Locations of regions of interest used for the analysis of default mode network connectivity in rat brain fMRI; slice thickness 1 mm. ROIs are overlaid on the reference brain, which was acquired with a functional spin-echo echo planar imaging sequence. Numbers located under individual brains indicate distance from the bregma in mm.

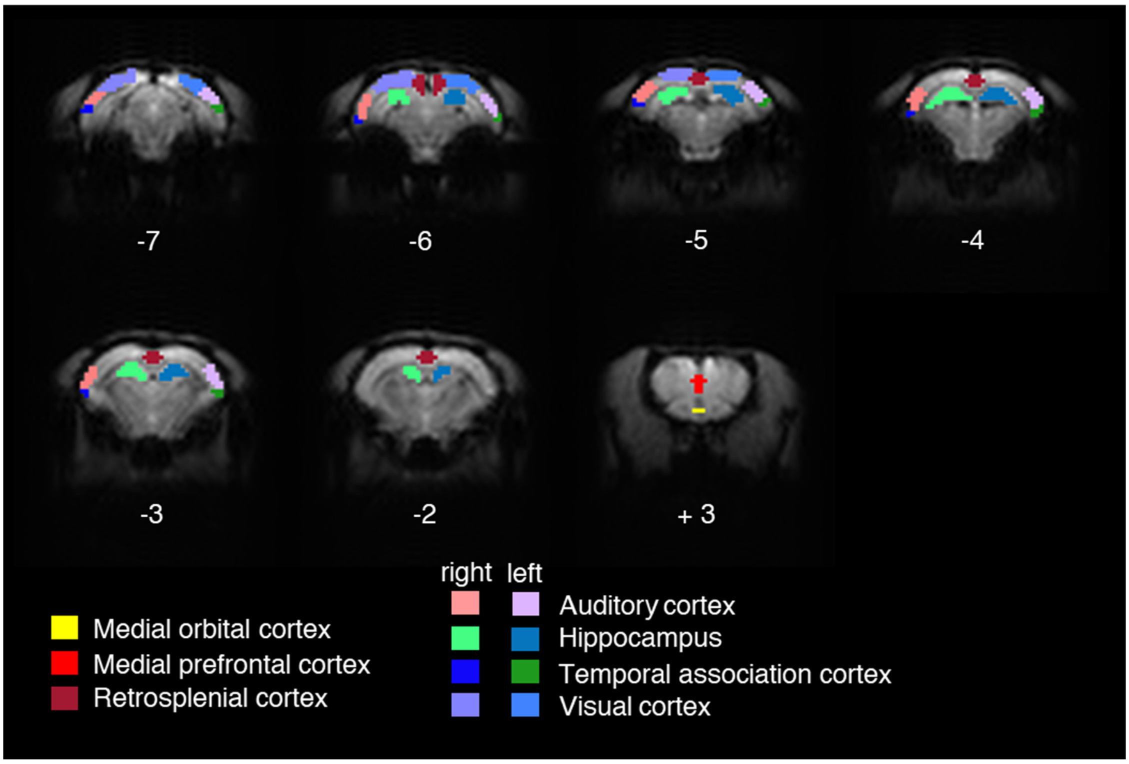

Figure S2 – Brain sections for analysis of optical density. Illustrative positions of Black-Gold II-stained brain sections selected for analysis of optical density in large white matter structures with indicated approximate distance from the bregma in the adult animal. Coronal brain sections from 15-, 18-, 28-, and 72-day-old rats are displayed.

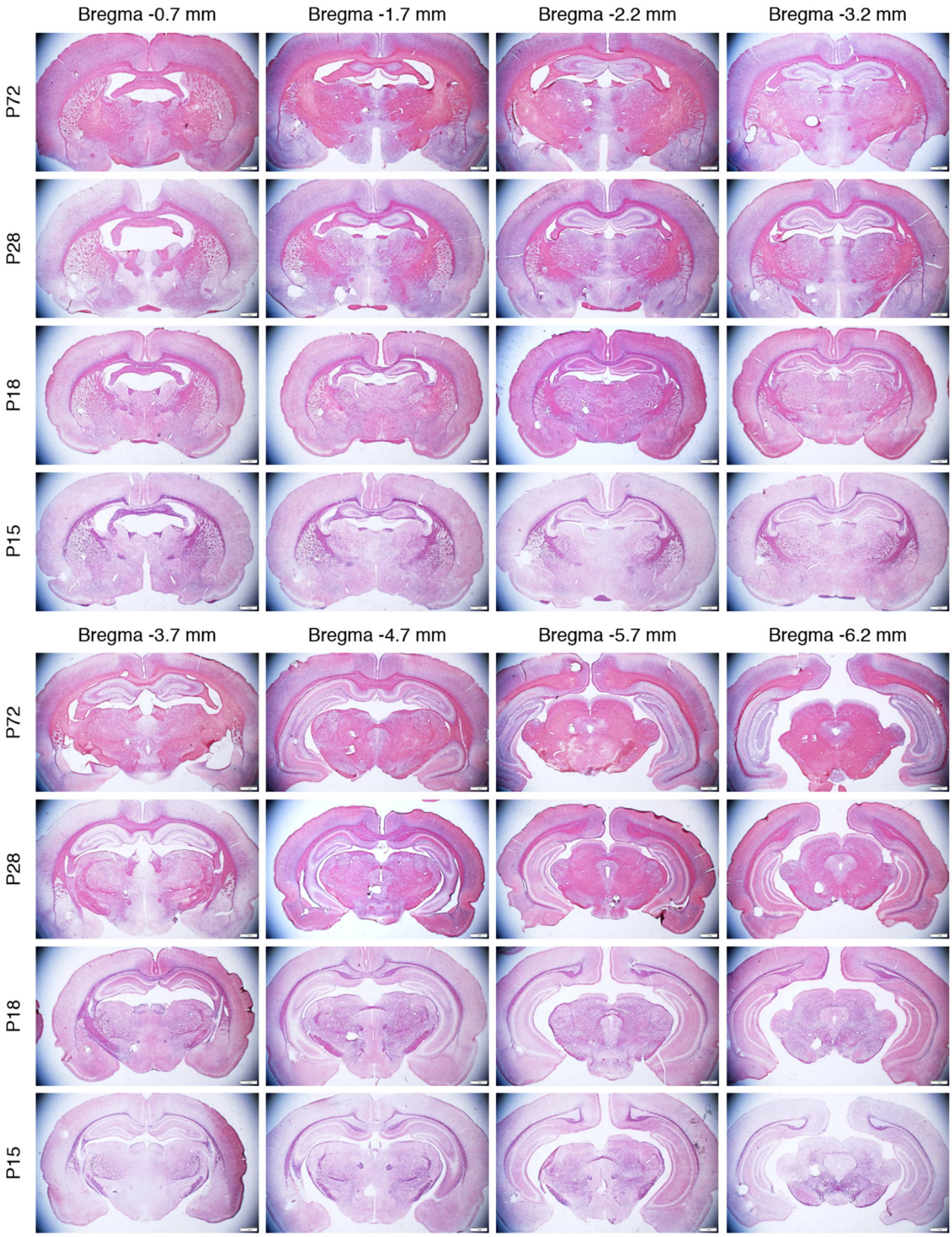

Figure S3 – Position of regions of interest (ROIs) for the optical density quantification. Representative positions of ROIs in white matter structures in left and right hemisphere

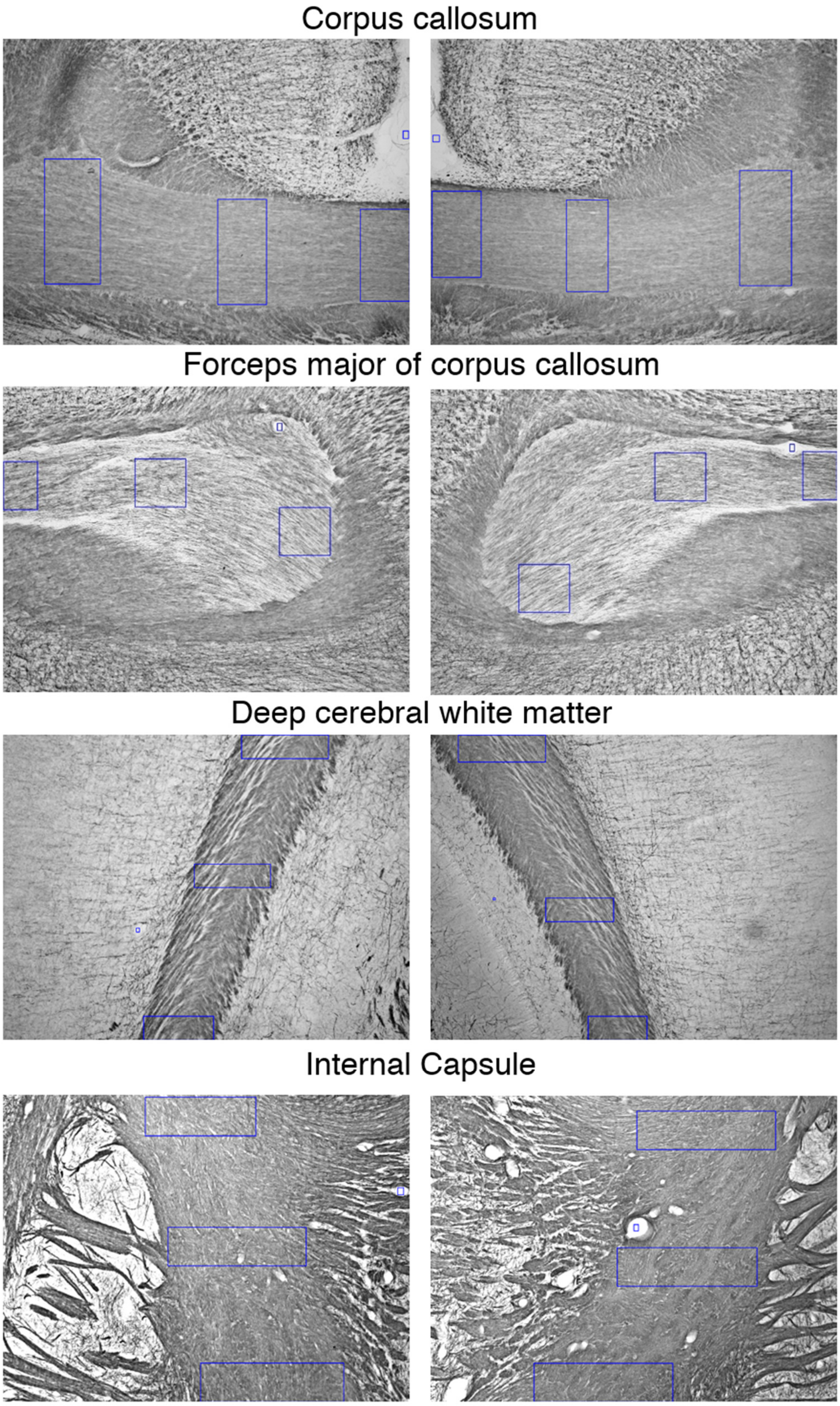

Figure S4 – Animal weight.

Daily weight percentage change (**A**) of rats following the induction of status epilepticus (SE) and controls (Ctrl). Daily follow-up is displayed across 16 days starting at the day of SE induction (0). The symbols represent the mean value and the error bars represent the mean standard deviation. The red asterisk (*) indicates significantly different values when comparing SE and control rats using Mixed-effects analysis. Displayed data include weight information from all animals used in the study (both MRI and histology groups). Graph (**B**) shows absolute animal weight [g] on the day of MRI & fMRI data collection (postnatal day (P) 28, 72 and 75) for animals after SE and controls within the MRI group.

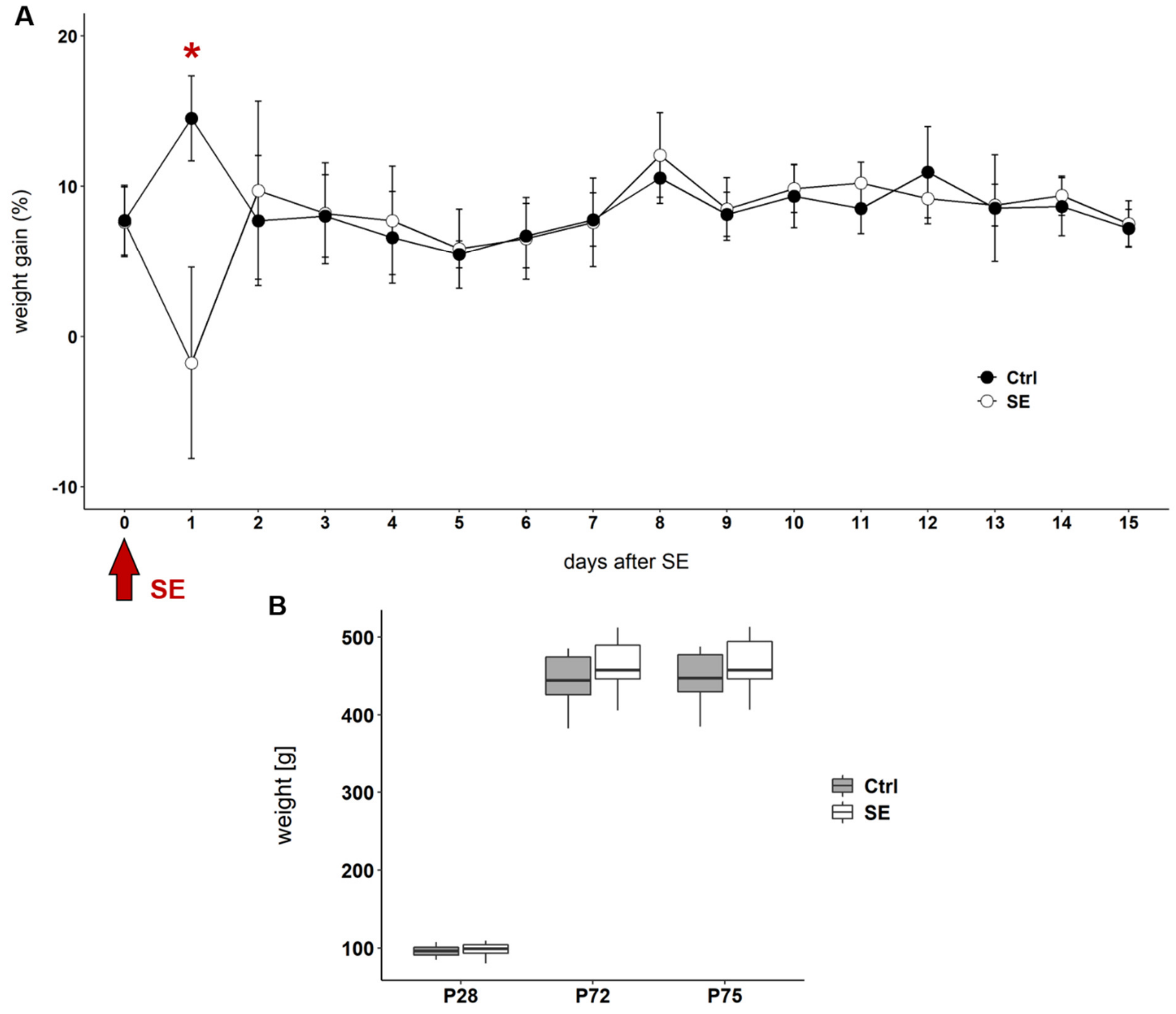

References

Conklin, P., Heggeness, F.W., 1971. Maturation of tempeature homeostasis in the rat. Am. J. Physiol. 220, 333–6.

Drachman, D., 2012. “Happy Trials to You” Adjusting for Multiple Comparisons.

Ranganathan, P., Pramesh, C., Buyse, M., 2016. Common pitfalls in statistical analysis: The perils of multiple testing. Perspect. Clin. Res. 7, 106. https://doi.org/10.4103/2229-3485.179436

White, T., van der Ende, J., Nichols, T.E., 2019. Beyond Bonferroni revisited: concerns over inflated false positive research findings in the fields of conservation genetics, biology, and medicine. Conserv. Genet. 20, 927–937. https://doi.org/10.1007/s10592-019-01178-0
